# What teleology (if any) does the Organisational Approach naturalise?

**DOI:** 10.1007/s40656-026-00731-8

**Published:** 2026-04-17

**Authors:** Rebecca Riccardo Cuciniello

**Affiliations:** Dipartimento di Antichità, Filosofia, Storia (DAFIST), Philosophy Section, University of Genoa, Via Balbi 4, 16126 Genova, Italy

**Keywords:** Biological organisation, Development, Teleology, Mechanism, Design, Epigeneticity, Kant

## Abstract

The Organisational Approach (OA) draws from Kant’s notion of natural purpose to offer a naturalistic account of teleology grounded in biological organisation, and a characterisation of biological purposiveness, i.e. what goals organisms have, and how they seek and achieve them. This paper re-examines the OA in light of two persistent issues: (1) whether organisational teleology can be causally realised in a way compatible with mechanism; and (2) how far the OA successfully extends across biological areas, development in particular. I distinguish two versions of the OA: a *minimal model*, focused on the self-maintenance of a stable organisation; a *regulative* model, which introduces regulation as a principle of teleological change. I argue that the minimal model successfully naturalises teleology, but at the price of a too narrow view of purposiveness as physiological self-maintenance. Instead, the regulative model offers a *sui generis* and potentially fruitful view of teleological change, blurring the boundary between physiology and development; however, it struggles to remain a naturalistic account. I introduce “design” and “epigeneticity” criteria for biological teleology to show how different ways of defining biological goals can either hinder or favour teleological naturalism, and thus the OA itself. This paper thus clarifies what kind of teleology the OA can naturalise, and where further theoretical work is required.

## Introduction

Recent works in the theory of biological autonomy have advanced a view of organisms as self-determining natural agents, demonstrating a great range of applicability across areas of biology (Barandiaran & Etxeberria, 2026; Bich 2024a; Moreno & Mossio, 2015; Mossio, 2024; Ruiz-Mirazo & Moreno, 2012; Virenque & Mossio, 2024). At the core of biological autonomy theory is the Organisational Approach to biological teleology (OA), a framework explicitly inspired by Immanuel Kant’s *Critique of the Power of Judgment* (CPJ; 1790) and centred around the notion of “closure of constraints” (Montévil & Mossio, 2015; Mossio & Moreno, 2010). The OA claims to provide (1) a *naturalising* causal account of teleology which is both scientifically grounded and compatible with mechanism, and (2) an appropriate characterisation of biological purposiveness, i.e. what organisms’ goals are, and how organisms pursue them (Bich 2024b; Mossio & Bich, 2017).^1^

The history of the debate on teleology has long been shaped by Kant’s “controversial legacy” (Gambarotto & Nahas, 2022). Within this legacy, two difficulties affecting the OA’s claims (1) and (2) have emerged.

The first concerns the *causal realisability* of teleology i.e. whether teleological relations can obtain within causal mechanistic systems. While Kant first characterised “organised and self-organising being[s]” (CPJ, p. 374) as natural purposes, he also denied – against the OA’s aspirations – that teleology could be naturalised. Recent critics still echo Kant’s own *caveat*, particularly regarding the problematic causal status of the organism as a self-determining whole (DiFrisco & Gawne, 2025; Teufel, 2011).

The second issue concerns the scope of applicability of the OA across biological areas. Huneman’s (2014, 2017, 2024) Kantian approach to contemporary biology has highlighted the epistemic role of various forms of teleological thinking in physiology, developmental, and evolutionary biology, but has also shown how these render teleology increasingly problematic. The question is whether the OA offers instead a teleological naturalism for all such areas or, as some critics have argued, it is still a too narrow view of teleology (Nuño de la Rosa, 2010; Rama, 2025a).

This paper sets to re-examine the OA in light of these two interconnected issues. I distinguish between two versions of the OA: a minimal model focused on the self-maintenance of a *stable* organisation (Montévil & Mossio, 2015; Mossio et al., 2009; Mossio & Bich, 2017); a regulative model introducing regulation as a principle of organisational *change*, specifically in adaptivity and development (Bich 2024a, 2024b; Bich & Skillings, 2024).^2^ Thus, I argue as follows. First, the OA’s naturalisation strategy, specifically devised for the minimal model, successfully reconciles teleology and mechanism, but pays the price of a narrow view of biological purposiveness. Second, the regulative model articulates a *sui generis* view of biological purposiveness antithetical to that assumed by its critics, blurring the boundary between physiology and development. However, the regulative model faces significant challenges in remaining a naturalising account.

In the following Sect. 2, I will revisit Kant’s problem of the causal realisability of teleology, and some recent reformulations. Section 3 draws from Huneman’s work on design and epigeneticity to discuss how different conceptions of teleology across biological sciences can either hinder or favour teleological naturalism. Section 4 focuses on the minimal model, while Sect. 5 focuses on the regulative model, particularly its account of development, possibly the OA’s most problematic area of application so far. Section 6 highlights remaining difficulties. Far from unreservedly defending the OA, this paper examines its theoretical advancements and limits, while also outlining directions for further work.

## The problem of causal realisability

The organisational tradition draws from Kant’s work to describe organisms as purposive “Kantian wholes” (Kauffman, 2021), capable of self-production, or *autopoiesis* (Maturana & Varela, 1980; Weber & Varela, 2002). The OA builds on this line of thought. Kant famously defined natural purposes as “organised and self-organising being[s]” (CPJ, p. 474) in terms of *parts/whole causal relations*.^3^ As a preliminary observation, it is important to recall that Kant framed purposiveness in these terms as a way of translating means/goal relations beyond the sphere of intentionality (Illetterati, 2008, 2014). An entity is purposive when its parts are *for* something (means to a goal), produced according to an *idea* of the whole which normatively predetermines the conditions of its own realisability (as with artefacts).^4^ The crux, for Kant – and for the OA as well – is whether such parts/whole relations can obtain within mechanistic nature.

Kant conceptualises natural purposes in the *Analytic of Teleological Judgment* in two related ways. In § 64, a natural purpose is “is *both cause and effect of itself*” (CPJ, p. 370). This apparent self-causation is exemplified by phenomena of reproduction, development and self-maintenance, and reciprocal dependence between an organism’s parts (p. 371).^5^ § 65 offers further elaboration. First, “the possibility of its parts (as concerns both their existence and their form) must depend on their relation to the whole” (p. 373). This expresses Kant’s general notion of purposiveness as *organisation* (Ginsborg, 2004; Huneman, 2014, 2017; Illetterati, 2008), shared by organisms and artefacts. Second, for purposiveness to be natural, specific circular relations between parts, and the parts and the whole must obtain. Parts-to-whole dependence involves reciprocal interactions between parts which, by *producing* and *shaping* each other, also constitute and maintain a whole (CPJ, pp. 373–374). Conversely and reciprocally, this whole also determines “the form and combination of all parts” (p. 373). Once the parts/whole “loop” is closed, we have an “organised and self-organising being” (p. 374), wherein “*everything is a purpose and reciprocally also a means*” (p. 376).

Kant believes that this kind of whole-to-parts dependence can only be grasped by our teleological judgment. Natural purposes are thus the occasion for the antinomy between two maxims of judgment, presented in the *Dialectic* section. The thesis states that “[a]ll production of material things and their forms must be judged to be possible in terms of merely mechanical laws” (CPJ, p. 387). The antithesis counters that “[s]ome products of material nature cannot be judged to be possible in terms of merely mechanical laws. (Judging them requires a quite different causal law-viz., that of final causes.)” (CPJ, p. 387). Mechanism in the CPJ has been variously interpreted – from strictly parts-to-whole causality (McLaughlin, 2001, 2014) to a Newtonian view of nature explaining natural products by the behaviour of matter under physical laws (Ginsborg, 2004, 2006). What matters here is that mechanism does not license teleological whole-to-parts dependence, preventing the parts/whole circularity by which something can be cause and effect of itself; in other words, teleology cannot be *causally realised* within a mechanistic worldview.

Recent critics of teleological and agential talk in the philosophy of biology share Kant’s own suspicion against whole-to-parts causal dependence. DiFrisco and Gawne (2025) argue that recent agential perspectives (see Sultan et al., 2022; Walsh, 2015; Walsh & Rupik, 2023) are mistaken in attributing to the organism as a whole causal control over behaviour, development, or self-maintenance. Phenomena affecting the whole organism (e.g. circadian rhythms, yeast cell cycles, escaping predators) are always controlled by specific parts (e.g. neurons, genes, various subsystems). The apparent teleological dependence of the parts on the whole reduces to mechanistic dependence between parts: “To say that it is ‘the whole system’… that regulates or controls some process… is not a well-formed causal statement” (DiFrisco & Gawne, p. 148) Similarly, Potter and Mitchell (2024) argue that mechanistic “causal holism, on its own, is not sufficient to attribute agency or ‘executive control’ to the developing organism” (p. 135) and thus cannot confer a “purposive dimension” (*ibidem*) to any biological process.^6^

Interestingly, these critiques largely spare the OA; Di Frisco and Gawne even concede that the OA may be a “defensible form of holism” (2025, p. 148), even though they sweepingly downplay the OA’s teleological connotations. Instead, Teufel’s (2011) critique of McLaughlin’s (2001) work on teleological functions (which anticipates in many respects the OA; see Mossio et al., 2009, p. 837) constitutes a sharp reprise of Kant’s *caveat* on naturalisation, and thus an important (but so far unaddressed) obstacle for the OA.^7^ According to Teufel, what makes teleology irreconcilable with mechanism is two *mereological* constraints within mechanism already at play in Kant’s CPJ: (i) identity is compositional, so if a whole’s token parts change, the whole does not remain the same; (ii) parts/whole asymmetry, i.e. parts can cause the whole *synchronically* because they *compose* it, but not vice versa. Accordingly, any teleological naturalism seeking compatibility with mechanism must satisfy three requirements.

1. Mereological requirement: the whole-as-cause should precede in time the parts-as-effects; Teufel calls this *diachronic* “downstream” causation.
2. Teleological requirement (reflexivity): because a circular relation must hold between parts and the whole through time, the whole-as-cause at t_1_ and the whole-as-effect at t_2_ should remain the same.
3. Causal requirement: we should avoid backward causation, where the whole is simultaneously cause and effect of its own effects.

Given these requirements and the above-mentioned constraints, Teufel argues that any teleological naturalism cannot be mereologically, teleologically, and causally coherent at the same time. Satisfying mereological and teleological requirements leads to backward causation: because the whole at t_1_ and t_2_ are the same, it is at once cause and effect of its own effects (the parts). Satisfying mereological and causal constraints sacrifices reflexivity: “a whole can have effects on the parts and their properties… so long as the whole that does the causing and the whole composed by the (now) changed (or even ex-changed) parts succeed each other in time (*diachronically*) and are thus neither *temporally* nor *compositionally* quite *the same*” (Teufel, 2011, p. 257). This leaves us with downstream whole-to-parts causation, which, without reflexivity, “walks and quacks like good old mechanism” (*ibidem*).^8^ Finally, satisfying causal and teleological constraints – synchronous reflexivity without backward causation – means giving up mereological requirements i.e. defining the whole-as-effect mereologically, but the whole-as-cause non-mereologically, as something other than the compositional sum of its parts.

Teufel’s analysis thus highlights two problems for teleological naturalism. One is backward causation, of which Teufel distinguishes two flavours. “*Temporally* backward” backward causation involves the traditional problem of final causes acting “from the future.” “*Causally* backward” backward causation, instead, involves something being both cause and effect of its own effects (regardless of the arrow of time), and thus, of itself. Perhaps, as Kant seemed to suggest in § 64, we should embrace self-causation; the dilemma, of course, is how to do this naturalistically. Another problem, on which I want to focus, concerns the *identity* of the whole as both cause and effect *and* through time. We can better understand its teleological relevance by recalling how, already for Kant, whole-to-parts dependence was never meant to *mirror* parts-to-whole causal-compositional dependence, but to express in non-intentional terms means/goal relations. As Walsh (2012, 2015, 2017, 2018) has extensively argued, this means/goal relation is one of counterfactual dependence or invariance: a goal variously determines its means so it can be invariantly achieved across a range of counterfactual circumstances. This is unproblematic for artefacts, where goals are external to their means i.e. parts depend on an external idea of the whole in the mind of a maker. When it comes to organisms, which *intrinsic* biological goal is invariantly achieved through both material and formal changes such as metabolism, self-repair, or development? In other words, how does the whole as cause and effect maintain its *identity* even as its parts change?

On this note, Teufel suggests that teleological naturalism might work if a naturalistic but non-compositional account of the identity of such “Kantian wholes” is provided, allowing a system to change its parts while remaining the same *stricto sensu* (and not heuristically), thereby achieving reflexivity. However, this would require abandoning the mereological talk of parts/whole relations central to Kant’s own *Analytic* or formulating an “organism-friendly mereology” (p. 260). He dismisses both paths as “non-starters… The empirical reality of ‘intrinsically purposive’ entities or processes in nature is a myth” (*ibidem*). It remains to be seen whether the OA can evade these difficulties concerning both self-causation and whole-identity, while remaining an *organisational* account, essentially grounded in parts/whole analysis.

## Biological teleology is said in many ways

In the last decades, a growing number of works has called for a comeback of teleology in contemporary biology. Some have maintained, following Kant, that while biological sciences can produce complete mechanistic explanations, we still need a *heuristic* “teleological outlook on nature in order to be able to think of ourselves as investigating, for instance, a tree or a bird’s eye” (Breitenbach, 2009, p. 53), rather than mere physical aggregates (see Breitenbach, 2006, 2008; Desmond & Huneman, 2020; Lotfi, 2010; Quarfood, 2006; Zammito, 2006). Others have called for a *naturalising* approach (Nahas & Sachs, 2023), whereby teleology retains explanatory validity and autonomy from mechanism, without referring to distinct *explananda*: “For some events an adequate understanding of their place in the natural world requires that their occurrence really is explained twice over: once by appeal to the mechanisms that cause them and once by appeal to the purposes they subserve” (Walsh, 2012, p. 174). Among these, the OA stands out as it treats teleology not only as a necessary *explanans*, but as an *explanandum* as well (Bich, 2024a, p. 16). What these positions share, however, is a perception that organisms cannot be fully explained by mechanism alone. In particular, as Ginsborg (2004) put it, organisms display a twofold “mechanical inexplicability”: we cannot fully explain (i) the form and configurations that organisms take, and (ii) their physiology, development, and behaviour, by treating parts in isolation from the whole. This is not to say that we cannot explain, say, digestion based on what stomach, intestines etc., do. Rather, parts are no more “basic” than the whole, their existence and properties depend on it. Accordingly, biological explanations should be “non-reductive and, in the end, circular; what accounts for the powers of the organism is ultimately the organism itself (or another organism of the same species)” (p. 47). It is this twofold parts/whole apparent non-separability that teleology interrogates in two ways: (i) *what goals* do organisms have? And (ii) *how* do they seek and achieve them?

Yet, there are reasons to believe that contemporary biology, particularly with Darwin’s theory of evolution by natural selection, while it does make heuristic use of teleology, also systematically undermines it (Ginsborg, 2006; Hull, 1969), constituting an ulterior obstacle to teleological naturalism. As I am going to argue, whether biology forecloses teleology or not depends on how goals (i) and goal-directed activities (ii) are conceptualised, offering a preliminary characterisation of the kind of teleology that can be naturalised by the OA.

Huneman (2014, 2017, 2024) has approached this topic speaking the language of Kant’s *Analytic*, distinguishing two criteria for biological purposiveness, *design* and *epigeneticity*. Design and epigeneticity capture two aspects of parts/whole teleological relations, respectively: (i) whole-to-parts dependence, which defines organismal goals, and (ii) parts/whole circularity, which focuses on how organisms realise certain goals. Huneman’s central claim is that design and epigeneticity have served as epistemic guides respectively in evolutionary and developmental biology; these, however, have also undermined parts/whole non-separability, making the plausibility of a “Kantian stance in a Darwinian world” (Huneman, 2017, p. 379; see 2024, p. 133) ever more problematic.

In evolutionary biology, already since Darwin (Gardner, 2009), design has expressed the idea that “organisms are wholes in which parts fit the needs and demands of the persistence of these wholes” (Huneman, 2024, p. 235). This of course involves both *functional integration* (the effects of each part cohere towards a goal) and *differentiation* (each part provides different but complementary contributions to goal-attainment). In an evolutionary perspective, particularly that of the Modern Synthesis, this means to say that organisms are “bundles of adaptations” (Huxley, 1942, p. 420). The logic of design is particularly at play in *adaptationism* (Gould & Lewontin, 1979), a habit of thinking prevalent in fields like palaeontology, functional morphology, or behavioural ecology (Huneman, 2019). The adaptationist method asks for each individual trait: *what is it for?* – a teleological question (i). The answer, in turn, reconstructs the selective pressures that rendered the (heritable) trait optimised for trait-fitness and stable across generations. Importantly, selective pressures involve both the environment and other traits of the trait-bearer organism, creating trade-offs between selective “competing demands” (Gould & Lewontin, 1979, p. 585). Through this process, natural selection produces functionally integrated and differentiated wholes adapted to their environment. Though Gould and Lewontin criticised adaptationism for “atomising” “the organism into independent traits, forgetting the “integrated whole” within which they first emerged, its logic remains teleological *sensu* design criterion or whole-to-parts dependence. Each trait’s form and persistence (after random appearance) depend on a prior principle of “systematic unity”^9^ (a “whole”), namely natural selection: “the external world poses certain well defined ‘problems’ for organisms… whose morphological, physiological, and behavioural traits represent the best *solutions to the problems*” (Lewontin, 1983, p. 97; my emphasis).

I call this a *stronger* interpretation of design, whereby biological goals *qua* adaptations describe a determine normative idea to which individuals may conform to or not. For instance, a viable goat born with two legs would be considered defective, since the norm, for goats, is to have four legs. In philosophy of biology, this logic is at play in etiological or selected-effects accounts of teleological functions, where organisms are (or appear^10^) purposive because they are *designed* by natural selection (Garson, 2016, 2019, Garson and Papineau 2019; Haig 2020; Millikan 1989; Neander 1991a, b; Rifkin and Garson 2023). Here function and malfunction ascriptions ultimately rely on comparing a part’s activity to a norm, namely its evolutionary history, or rather, our reconstruction of it: parts are functional if they do what (we think) they were selected for.

Epigeneticity, in turn, defines the particular teleological outlook guiding developmental biology, which treats developmental processes as goal-directed (ii).^11^ Huneman focuses specifically on recent evo-devo research on how gene-expression is epigenetically regulated depending on within-organism and organism-environment interactions.^12^ He takes gene regulatory networks as displaying a kind of Kantian parts/whole circularity, where gene-effects on differentiation, morphogenesis, or cell functioning are both context-dependent and expected to produce certain phenotypic goals. For instance, the cellular targets of apoptosis during organogenesis are intelligible only by “assum[ing] the whole organism as a norm for pattern formation” (Huneman, 2017, p. 383). More generally, in developmental biology, “concepts [of the whole] that take the role of norms in biological inquiry behave like *attractors*” (Huneman, 2024, p. 132): developmental processes are expected to invariably achieve and maintain certain configurations even as initial conditions change.

This apparent circularity in developmental biology is often underwritten by evolutionary assumptions. As Huneman notes, in many research programs that centre on genes and genomes, what justifies the developmental biologist’s expectation of a certain pattern or phenotypic outcome is the very adaptationist idea of design. Development is seen as highly *canalised* towards certain configurations because determined, in turn, by a genetic program evolved by natural selection (Huneman, 2017, p. 386). This, of course, is the traditional idea of *teleonomy* in philosophy of biology (Dresow & Love, 2023; Mayr, 1974, 1998; Vitale, 2022). In a teleonomic perspective, biological processes (development, behaviour etc.) appear goal-directed because genetically programmed. In particular, the context-dependency of gene-effects is interpreted it in terms of an evolved *norm of reaction*, i.e. a genotype’s ability to express determinate phenotypes depending on internal and external environments (Pigliucci et al., 1996).

Thus, we can see how under a stronger interpretation of design – what we think parts *should do* – the parts/whole non-separability that seemed to require a teleological outlook is dissolved. First, as genes assume causal primacy over developmental processes (Austin, 2015), parts do not depend on the whole they are part of. The circularity described by the epigeneticity criterion is thus broken, and “Life’s just one damned bit of DNA after another” (Teufel, 2011, p. 257). Second, because the genetic program can “stand in” for the whole because it is evolutionarily designed, its purposive-like character assumed by teleonomy is subordinate to natural selection. Finally, there are reasons to believe that the heuristics of epigeneticity and design do not always apply to developmental and evolutionary biology. In developmental biology, many developmental changes are understood to be stochastic in nature, or regulated only at the local level, thus being independent from the whole.^13^ In populations genetics, adaptationism predicts that fitness is always maximised (e.g. via inclusive fitness, or evolutionary stable strategies following variation-dependent selection); but this is not always the case (Christie et al., 2022; Okasha, 2022). Once we abandon adaptationist illusions of fitness maximisation and evolutionary progress, organisms appear merely as complex mechanisms, shaped by a contingent evolutionary history, which teleology only contributes to obscuring rather than illuminating, by describing organisms as something they are not. In sum, the fact that both evolutionary and developmental biology begin with a teleological question (“what is this trait for?”; “what is this process directed at?”), and end with a mechanistic explanation (“it was selected for…”, “it is programmed…”) signals less a vindication and more an *end* of teleological (let alone Kantian) thinking in today’s biology (Ginsborg, 2006; Hull, 1969).

And yet, teleology is making a comeback. Recent works in evolutionary theory and evo-devo have questioned both the assumptions that organisms are passive products of selection (strong design), and that developmental outcomes are essentially “scripted” in the genotype’s norm of reaction (design-based epigeneticity). Accordingly, by depicting both evolution and development as organism-guided, some researchers have called for an agential view of organisms, controlling their own development and shaping their own evolution (Aaby & Desmond, 2021; Jaeger, 2024; Lala et al., 2014, 2019; Nadolski & Moczek, 2023; Newman, 2023; Sultan et al., 2022; Uller & Lala, 2019; Walsh & Rupik, 2023; Walsh & Sultan, 2024). This suggests that an alternative interpretation of design and epigeneticity, that is to say, of goals and goal-directed activities is possible.

A weaker interpretation of design would not treat the “whole” within whole-to-parts dependence as an evolved norm to which to compare individual organisms; rather, it would take it as no more no less than the configuration parts themselves actually realise in individual organisations. On this view, a four-legged and a two-legged goat are on par, since their parts depend on the four-legged and two-legged wholes respectively (in fact, with important adaptive differences; West-Eberhard, 2003). The epistemic role played by the whole here is much more liberal, in that parts are expected to contribute to the whole *whatever its form*; in other words, “[*v*]*iability is a norm*” (Huneman, 2024, p. 131).^14^ Whether physiological or developmental processes are strongly canalised or not does not matter for teleology; the emphasis shifts from robustness to adaptive plasticity i.e. the organism’s capability to produce functional variations and novelties to maintain viability, suggesting that goals are irreducible to classic adaptations (Moczek, 2022; Newman, 2023; *contra* DiFrisco & Gawne, 2025; and Potter & Mitchell, 2024). Here, therefore, epigeneticity – what organisms do – has primacy over design; design is in itself contingent on the activity of the parts, which nonetheless realise a circular relation with the whole.

As the remainder of this paper will show, while the OA is in line with this possible interpretation of design and epigeneticity, it still faces challenges in translating it into a full naturalising account.

## The Organisational Approach: why the minimal model works

Despite being rooted in the “inherent connection between self-determination, teleology, and biological organisation… traced back to Immanuel Kant’s *Critique of Judgment*” (Mossio & Bich, 2017), the OA sets to *naturalise* biological teleology where Kant took it as a heuristic for mechanistic explanations. This section focuses on what I call the OA’s minimal model, centred on the self-maintenance of a stable organisation.^15^ I defend the OA’s claim that biological organisation, as closure of constraints, grounds teleology as a “legitimate and admissible conception of causality from the standpoint of natural science” (2). As seen in Sect. 2, scepticism about teleology largely stems for the causal improbability of the “organism as a whole” within whole-to-parts dependence, an essential element of any attempt to naturalise a Kantian kind of teleology. I begin by outlining the distinction between processes and constraints within biological organisation (4.1), and how closure of constraints realises self-constraint (4.2). Afterwards, I discuss the OA’s naturalisation strategy more closely (4.3), and how purposiveness is understood in the minimal model (4.4).

A couple of disclaimers. The OA claims that biological organisation grounds teleology as well as functionality and normativity. In what follows, I will not address the OA in the broader debate on biological functions (García-Valdecasas and Deacon 2024; Mossio et al. 2009; Mossio and Pontarotti 2019; Mossio and Saborido 2016; Rama 2025b; Saborido et al. 2011; see Artiga & Martínez, 2016; Garson, 2017 for critiques). Nor will I defend the OA’s view of normativity as counterfactual dependence of an organism’s existence on its properties and activities – a position still contentious (Corti, 2022, 2023) but under development. My concern is to outline the central features of organisational teleology in the minimal model, of which functional integration and differentiation are essential aspects. While much of this section might appear as a retelling of already established organisational principles, my analysis brings to the fore, in novel terms, *what makes the minimal model work*, particularly against so far unaddressed problems (cf. sections 2–3), while also establishing the tools to show how the regulative model does *not* work (see sections 5–6).

### Processes, constraints, closure

The core thesis of the OA is that biological organisation realises teleology because it is an *inter-level causal regime* characterised by thermodynamic openness and organisational closure *qua closure of constraints*.

Drawing on Piaget (1967), Kauffman (2000), and Rosen (1985, 1991), the OA distinguishes two kinds of causes: *processes* and *constraints*. Processes are material (physical or chemical) transformations involved in the production, maintenance, or destruction of entities. Constraints are entities that (i) act on processes without being affected by them, remaining locally stable at the relevant timescale, and (ii) *constrain* such processes influencing the production etc. of the output entity (Montévil & Mossio, 2015).

Because relations of dependence between constraints (e.g. C_2_ on C_1_) are mediated by the processes they constrain, they are inherently generative: C_1_ produces C_2_ by constraining a process resulting in C_2_, or more precisely, constraining C_2_ *as a process*.^16^ The difference between processes and constraints is a matter of timescale: the same entity can be both, depending on whether we consider its maintenance and transformation (process) or its causal efficacy over other processes (constraint). For instance, an amoeba’s outer membrane acts as a long-lived constraint over exchanges with the environment, yet it is also a process insofar as it is continuously produced and repaired by its molecular components. Crucially, dependencies between constraints occur *across* timescales: they can be *slow* (C_2_ is dependent on a more stable C_1_ at a higher timescale), or *fast* (C_1_ is dependent on the repeated activity of C_3_s at a lower timescale).

Given this distinction, we can now see how biological systems, being thermodynamically open and organisationally closed, can thereby self-maintain. They are thermodynamically open because they are traversed by a continuous flow of matter and energy sustaining all processes through which each constraint, and the whole organisation, is produced. Organisational closure, instead, specifies how constraints are related so as to constrain such thermodynamic flow into maintaining the system, i.e. how biological organisation achieves *metabolic self-maintenance* (Bich, 2024a).

Closure is realised when, given a set of constraints, each constraint C_1_ depends on at least one other constraint of the same set C_2_, and at least one other constraint C_3_ depends on C_1_ (Montévil & Mossio, 2015; Mossio & Moreno, 2010). In Kantian terms, under closure, constraints are both cause and effect of each other with respect to their existence – dependences are generative – and their form – constraints reciprocally *specify* the conditions of their activity (Mossio & Bich, 2017). Constraints under closure are thus mutually dependent and mutually constraining.

Moreover, closure requires that constraints contribute to the system in different but complementary ways. They must be functionally integrated and differentiated – whereby a constraint’s function is defined as its *causal* contribution to an organised regime of self-maintenance in which it is itself produced and differentiated (Mossio et al., 2009; Mossio & Moreno, 2010); in other words, “Closure of constraints is… closure of functions” (Moreno & Mossio, 2015, p. 71). First, constraints cannot all have the same properties or perform the same roles: this would not only foreclose the complexity of real biological systems but, crucially, also preclude the inter-level, cross-timescale relations that enable self-maintenance (constraints acting on different processes at different timescales). Second, while differentiated, constraints need to jointly contribute to each other’s functioning as well.

This double requirement for constraints – differentiation and integration – is captured by the notion of *functional presuppositions* (Bickhard, 2000; Christensen & Bickhard, 2002; Moreno & Mossio, 2015; Saborido & Moreno, 2015). Although the term “presupposition” often carries ad hoc normative connotations (see Saborido et al., 2016; Saborido & Moreno, 2015, on malfunction and pathology), it captures what mutual dependences consist of. Each constraint’s “structure… is such that it ‘presupposes’ that it will enter in a regime of interactions not only allowing a generic viability, but also the appropriate regime of interactions” (Saborido & Moreno, 2015, pp. 91–92). The function of a constraint C_1_, in turn, is to satisfy the functional presuppositions of its dependent constraints C_2_ (Bickhard, 2000); that is, to contribute to their activities in ways that also maintain its own operation within the whole. For instance, the heart’s functioning presupposes the lungs’ oxygen intake and the vascular system’s distribution of oxygenated blood, which in turn presuppose the heart’s beating at a certain rate.

Due to mutual dependences, constraints’ relations must remain the same through time. In the minimal model, closure appears as an inherently stable regime: to self-maintain, it must continuously meet its own endogenous conditions. Such a system has a limited dynamic range. When facing external perturbations, it displays relative flexibility or “dynamical stability”: perturbations might momentarily alter a constraint’s functioning, but mutual dependences allow the system to regain its initial state, “absorbing” the perturbation (Bich et al., 2016, p. 247). If a perturbation exceeds the system’s tolerances, a constraint can *fail*, causing others to fail as well, like a domino (Christensen & Bickhard, 2002). Hence, in the minimal model, *persistence* through time means remaining *identical*.^17^ A minimal system like this cannot sustain qualitative organisational and functional changes, as they would break the circularity between constraints.

This is not to say that the minimal model excludes variability (Montévil & Mossio, 2015, p. 25). Mutual productive dependences alone do not always guarantee functional integration, given the system’s complexity and susceptibility to variation. Constitutive self-maintenance regimes require higher-level regulatory control, or second-order closure (Bich et al., 2016; Moreno & Mossio, 2015; Mossio & Moreno, 2010; Saborido & Moreno, 2015). Regulatory constraints, though sustained by constitutive ones, are *metabolically decoupled*, meaning their functioning is relatively independent from their production. As they are triggered by internal or external changes, they modulate other constraints’ activity rates, relations, and transitions between available regimes of self-maintenance. In short, the task of regulation is to guarantee functional integration (Bich & Bechtel, 2022a, b).

Still, in the minimal model, variability remains subordinate to a higher “invariance of closure” (Montévil & Mossio, 2015, p. 26). In light of such organisational invariance, lower-level variability is synonymous with the “contingency of biological systems” (*ibidem*) i.e. their susceptibility to noise, replication errors, environmental blows, and other perturbations to be dealt with to ensure functional integration. Regulation thus serves as a principle of *stability*. For instance, after losing a kidney, the remaining one hypertrophies to satisfy the functional presuppositions of dependent constraints (Saborido et al., 2016; Saborido & Moreno, 2015). In the minimal model, changes are *compensatory*, functional to the maintenance of certain organisational invariances.

In sum, the OA’s minimal model describes an inter-level cross-timescales causal regime of mutually dependent constraints that, by harnessing thermodynamic flow, maintain each other and the whole, enabling the continuous self-maintenance of a stable organisation.

### A Kantian look at self-constraint

The OA locates self-determination at the level of mutual dependences between constraints, realised as *self-constraint*. Unlike physical cycles, where circular relations exist between otherwise independent elements, self-constraint occurs when “the activity of a system, by producing some effects, contributes to specifying the conditions under which the circular relation as such can occur. It is in this precise sense that the connection between teleology and self-determination is to be understood” (Mossio & Bich, 2017, p. 1107).

To clarify in what sense self-constraint is seen as teleological, we can refer again to Kant’s parts/whole analysis: how does self-constraint “close the loop” between parts-to-whole and whole-to-parts dependence? As anticipated, natural purposiveness requires (i) mutual dependences between parts, by which parts (ii) constitute a whole, and (iii) are dependent on the whole itself.

Mutual dependence (i) takes place through and across timescales. Consider the reciprocal dependence between heart and lungs. Both are relatively stable, share correlated rhythms, thus acting as constraint at a timescale τ_1_. However, the heart-as-cause exists at a slower timescale than the lungs-as-effect, as it enables processes producing other constraints (e.g. lung cells) causing (and composing) the lungs at a faster timescale τ_2_, and vice versa. Reciprocal dependence between constraints requires both slow and fast dependences; parts that are cause and effect of each other, as cause *and* effect exist on different timescales.

The same reasoning can explain how parts constitute the whole (ii). The whole organisation, being thermodynamically open, is a sustained but transient process of production, maintenance, alteration, and eventual destruction. In fact, if we look at the whole organism *from beginning to end*, it is not stable – it undergoes the most radical change: it is “born”^18^, and it dies. In turn, the whole as a process is constrained by the activity of its parts, which, while cannot be more stable than the whole itself, can produce it through their continuous activity through time. Thus, when subject to closure, constraints *constitute* a biological whole because they *constrain* thermodynamic flow to maintain each other.

How do parts then depend on the whole (iii)? As Mossio et al. (2013) argue, Kant’s idea that “the existence of the constituents (the constraints) ‘depends on the whole’” simply means “‘depending on the whole network of interactions’… What is frequently described as a causal action of the whole system on its own constituents, in in fact the result of the interaction among hierarchically organised constraints (or subsystems of constraints)” (p. 18). The “whole,” in other words, is identical to the *relation* between constraints, what Montévil and Mossio (2015, p. 25) term the *invariance of closure*. To say that constraints depend on the whole amounts to say that they depend on each other.

Thus, the OA closes the loop between parts and whole: constraints subject to closure (i) constrain the whole as a process (ii), while their conditions of existence and activity are determined by and within the organisation itself (iii). In this way, the whole organisation, as a process, is maintained by the whole organisation realising self-constraint.

### The OA’s naturalisation strategy

How does the OA’s idea of self-constraint fare against the difficulties regarding the causal realisability of teleology presented in Sect.  2?.

DiFrisco and Gawne’s (2025) concern about the “organism as a whole” is easily addressed. They argue that attributing whole-organism control over internal properties or organism-environment interactions is causally problematic: any global effect can always be explained by *parts* of the organism. The OA, however, does not treat the organism as a causal agent acting on its parts. Rather, parts depend on and cannot be isolated from the whole network of interactions, as their causal powers are context-dependent and emerge from their mutual influence. In other words, the organisation as a whole can self-maintain and self-determine exactly because it is nothing more than the sum of its parts, provided of course that such a “sum” takes the form of an organisation.

But can the OA sustain a teleological naturalism, conciliating teleology with mechanism, given the difficulty of maintaining reflexivity, and therefore the identity of the whole through time and the identity of the whole as both cause and effect?

Teufel (2011) framed his critique to teleological naturalism as a problem of identifying a composite whole through changes in its parts, such that it can both exert diachronic causal influence on its components (licensed by mechanism, unlike synchronous downward causation), while remaining identical to those parts. Put differently, the problem is to account for parts/whole circularity *through time*. The OA pursues two strategies regarding the temporal dimension of closure. One *abstracts* from time (Montévil & Mossio, 2015, p. 19; Moreno & Mossio, 2015, p. 24; Mossio & Bich, 2017, p. 14; Mossio & Pontarotti, 2019, p. 13), doing away with diachronic vs. synchronic dependencies and emphasising timescales. The other reintroduces diachronicity through the notion of *organisational continuity* (DiFrisco & Mossio, 2020). I focus here on the first strategy, specifically devised for the minimal model; organisational continuity will gain relevance in the discussion of organisational changes later (see Section 6).

The *abstraction* strategy, inherited from Rosen (1985, 1991), considers a temporal interval wide enough for all constraints to exert their influences, then abstracts from the physical time in which they occur (the “before” and “after”). Thus, “the whole network of dependencies should be considered as one ‘block’ extended over multiple timescales” (Montévil & Mossio, 2015, p. 19). In abstract descriptions of closure, “there is no temporal ordering between [C_1_ and C_2_]; they differ uniquely with respect to the *orientation* of the dependence relation” (Mossio & Saborido, 2016, p. 8), upward and downward across timescales. This abstraction is not mere heuristic. Causally mutually-dependent constraints are not straightforwardly distributed in linear time e.g. C_1_-at-t_1_, C_2_-at-t_2_, C_3_-at-t_3_, and then again C_1_-at-t_4_, the latter C_1_ being non-identical to the former. Recognising the dual status of biological entities as both processes and constraints shows that (i) causal influence takes time, making causes inherently more stable than their effects, and (ii) constraints depend on more stable or repeated causes in turn. Thus, diachronic ordering misrepresents organisational causation: asking whether C_1_ acts “before or after” C_2_ assumes timescale parity.^19^ We could say instead that C_1_-to-C_2_ dependence is “synchronous,” not in the sense of an instantaneous influence at t_1_, but of a continuous influence at τ_1_. In turn, C_2_ can act as a constraint at the moment of its production, or later when needed; what matters is that, to the extent that it (indirectly) feeds into C_1_, C_1_ is already stable enough to enable C_2_.

The same rationale applies to parts/whole relations. AlreadyMcLaughlin (2001) proposed that, to reconcile parts/whole reflexivity with diachronic causation, we should “stretch” the whole through time, treating it as *the same* despite (or rather thanks to) changes in its parts. Thus, the whole-at-t_1_, influencing parts-at-t_2_, would be the same whole-at-t_3_, reconstituted by those parts. Teufel (2011) objects that this implies a non-compositional definition of the whole through time, which “capture[s] the identity of a ‘whole’ in name only” (p. 258). To show how the OA’s abstraction strategy avoids such nominalism, it is still useful to try to “unfold” closure through time. After all, biological systems do exist in time, they are not abstract entities.

All changes – the production of an enzyme, muscle contractions, or stomatal opening and closing in leaves – are *processes* of transformation and use of matter and energy that realise organisational features. Being thermodynamically open, the whole does not and *cannot* remain the same in a strict material compositional sense; living beings would not be *living* in any meaningful sense if they did.^20^ Yet self-constraint, and therefore parts/whole relations, occurs at a level above mere material composition. As extensively discussed, organisation self-maintains by constraining the thermodynamic process of its own material turnover. The whole-at-t_1_ influences its parts not as a sum of materials, but as a set of constraints, specifically their *relations*. The whole can influence its parts as a relational context within which the parts’ causal powers and effects are specified at each time. Then, the whole-at-t_2_, as an effect, is not merely a momentary aggregation of matter and energy, but the continued instantiation of the closed relations between constraints.

The whole, as both cause and effect, remains the same through time while remaining compositionally defined, that is to say, in a way compatible with mechanism. First, parts-to-whole *composition*, as in Kant, is enabled by the causal relations between parts, understood by the OA as productive dependences between constraints. These relations are compatible with mechanism, and in fact occur both in biological and non-biological systems (Bich & Bechtel, 2021, 2022b). Here though, what produces an emergent whole is the mutual dependence captured by closure. Second, the whole need not compose its parts to realise whole-to-parts dependence. The OA does not hypostasise the whole as an external agent but identifies it as an immanent relation between parts. This kind of composition is thus not material, but *relational*: it is constraints, not processes, that constitute the causally relevant whole. Perhaps, this might be the “organism-friendly” mereology floated by Teufel, where biological organisation, as a specific kind of causal system, is not *made of* matter, but of relationally defined entities, constraints subject to closure.

Finally, the OA’s abstraction strategy avoids the problem of “temporally backward” backward causation, as it does away with diachronic dependencies. Instead, it embraces parts/whole “*causally* backward” backward causation as self-causation, self-determination, and self-constraint: the OA offers a naturalised account of Kant’s claim that organisms are *cause and effect of themselves* (Mossio & Bich, 2017).

### Purposiveness in the minimal model

As I advanced in Sect. 3, any teleological naturalism should clarify what goals and goal-directed activities are, i.e. what biological features genuinely display parts/whole non-separability and can therefore be approached teleologically, as captured by the notions of design and epigeneticity. As I showed above, the OA makes parts/whole non-separability causally plausible. The remaining questions are what kind of teleology emerges (i), and what biological features it explains (ii).

First (i), the OA identifies the organisation’s goal or *telos* with the conditions of existence on which it exerts causal influences: “in the case of biological systems their goal and their own existence are one and the same thing” (Mossio & Bich, 2017, 1090); or, as Virenque and Mossio (2024) recently put it: “the purpose of living beings is their own organization” (p. 12). It should be noted that the OA sometimes identify the organism’s goal with its *existence in general*, sometimes with its endogenous *conditions of* existence.^21^ This might be a source of ambiguity, particularly when considering the teleology of *change*. If existence is the goal, organisational changes are goal-directed toward maintaining viability; if specific conditions themselves are goals, changes might alter goals without necessarily being goal-directed. However, as shown above (4.1), in the minimal model organisation is stable, and self-maintenance amounts to preserving certain core invariants. There is therefore no analytic difference between existence and the actual conditions thereof. Accordingly, in the OA’s minimal model *what* goals are, and *how* organisms achieve them are analytically equivalent: the organism’s goal – existence, or conditions thereof – is ultimately their very activity by which conditions are specified and maintained. As the OA often claims, organisms “are what they do” (Mossio & Bich, 2017); what they do is *being* (specifically, remaining identical through time).

It should not surprise then that the teleological (and functional) explanations licensed by the minimal model are thus as circular as the dependence between constraints themselves. As already Ginsborg (2004) put it, what accounts for the organism is the organism itself. Here, given closure of constraints, C_1_ mechanistically explains C_2_, which explains C_3_… which explains C_1_. Teleological explanations, instead, refer to the function of each constraint: C_1_ operates the ways it does *in order to* produce C_2_, more specifically, to enable C_2_’s causal contribution to self-maintenance (its functional role), and therefore to C_1_ itself.^22^ Thus, teleological explanations justified by closure refer to constraint’s contribution to self-maintenance: the organism acts the way it does *in order to* exist. For instance, mechanism explains how catabolite activator and lac repressor proteins modify *lac operon* expression and therefore metabolic regimes in *E. coli*, what triggers them etc. In turn, teleology explains that their activities are performed to maintain homeostasis and viability in different nutritional environments (Bich et al., 2016; Bich & Bechtel, 2022b). Appealing to goals thus does not refer to any mysterious “final causes,” but only to the circular network of mutual dependencies realising self-constraint.

In regard to point (ii), some authors suggested that this identification of goals and goal-directed activities (whose goal is their own maintenance), and thus the explanatory conflation of design and epigeneticity, describes the teleological profile of *physiology* (Huneman, 2014; Rama, 2026). This conflation differs from the Darwinian evolutionary reduction of epigeneticity to design seen in section 3. This was specifically at play in developmental biology, where developmental processes are explained by reference to a phenotypic outcome pre-specified in a designed genetic program. In physiology, instead, the organisation is already given, what matters is how it is maintained through metabolic and regulatory activities. This is what the OA’s minimal model focuses on. Its naturalisation strategy succeeds precisely because epigeneticity, the organisation’s purposive activity, “reduces to being integrated into a general design” (Huneman, 2014, p. 6) i.e. an invariant relation of mutual dependences. Under this reading, *the OA’s naturalisation strategy* (see 4.3) *is successful because the minimal model is a theory of physiology*.

This, however, implies a very narrow view of epigeneticity, where organisational relations, and thus goals, do not change, or if they do, they do so in a regulated manner among a set of available regimes, and to maintain certain invariants. When such relations do change, as in the case of development, it becomes problematic to identify a *self-*maintaining organisation. How, for instance, can a moth be cause and effects of itself if its caterpillar-stage conditions of existence and design differ so dramatically, without taking both as stages of the same teleonomic process of gene-expression, eventually foreclosing teleology (as per § 3)? How can we still claim that teleology is naturalised, and that the caterpillar and the moth are not the same *only heuristically* (as per § 2)? The next section moves from constitutive closure to biological regulation, addressing the OA’s applicability to development.

## Changing teleology: the regulative model

The recent update to the OA expands beyond self-maintenance, offering a richer view of biological purposiveness grounded in self-regulation (Bich, 2024b). Regulatory functions are already central to organisational accounts of many dimensions of physiological self-maintenance, such as integration and complexity management, minimal cognition (Bechtel & Bich, 2023; Bich 2018; Bich & Bechtel, 2021, 2022a, b), autonomy, agency, and behaviour (Moreno & Mossio, 2015; Virenque & Mossio, 2024), as well as health and pathology (Saborido et al., 2016; Saborido & Moreno, 2015). Regulated self-maintenance does not mean absolute lack of change: regulation is not just a “harmonising” principle, but a cause of adaptive change as well, controlling transitions between different regimes of self-maintenance, and therefore the *gain*, *loss*, and *change in available functions*. For example, recent organisational accounts of health conceive it as a mutable continuum rather than a fixed homeostatic balance (Bich & Menatti, 2025; Menatti et al., 2022).

As proposed by Bich (2024b, 2024a), regulated organisation does not simply *establish* and *achieve* its goals in the same activity but actively sets and pursues them across diverse scenarios. Unlike in the minimal model, where establishing and achieving goals are analytically equivalent activities performed by the same entities, now constitutive constraints achieve and maintain conditions of existence established by higher-level regulation, drawing on a repertoire of available functions, not operative all at once. What is important here is not that “it takes more” to stay alive (more levels of constraint, more control), but that biological purposiveness is a much more complex affair than achieving one invariant *telos*.^23^ Bich and Skillings’s (2024) recent treatment of development suggests a more radical and nuanced view of purposiveness, treating regulation as a principle of qualitative functional change, not subordinated to any higher invariant, signalling a significant theoretical departure from the minimal model. In this section, I first introduce the OA’s view of developmental purposiveness (5.1), then discuss critiques to the applicability of the OA to development (5.2). Here I do not mean to defend the OA against them but rather show how the updated OA takes development not as a *sui generis* biological process (compared to “normal” self-maintenance), but as paradigmatic of how organisms determine their own mutable *telos*.

### Non-intermediate *telē* and organisational continuity

Development is a “regulatory process that changes the number or types of functions of a regime of closure of constraints” (Bich & Skillings, 2024, p. 243), a qualitative change in the functional relations that make up organisation, by change in the relations between constraints (e.g. rates of activity), or change in the constraints themselves (e.g. loss and production of new organs). As such, it encompasses not only the formation of adult or reproductively mature forms, but also radical adaptive reorganisations (think of the human brain’s lifelong structural and functional changes). If in the minimal model purposiveness described a relational invariant, here it manifests as a kind of regulated variability.

In Bich and Skillings’ view, there are *no intermediate stages* in development. Consistently with the OA’s identification of the organisation’s *telos* with the conditions of existence it controls, development is not teleological because it is *directed towards* and end-state. As they argue, “adultocentrism” – defining development by its outcome as an adult or reproductively mature form – “is just a symptom; the real issue is the directional teleology that underlies it” (p. 257; see Minelli, 2011). Instead, development is purposive because it displays self-maintaining closure at each and all stages. *Development*, in other words, *is taken as a form of self-maintenance*. This view might appear counterintuitive. Take a tadpole developing into a frog (Bich and Skillings’ own example). If the tadpole’s intrinsic *telos* is self-maintenance, why do regulatory systems dismantle the “tadpole-closure” (with gills and tail) to leave space for the “frog-closure” (with lungs and legs)? In the minimal model, self-determination relies on circular relations between constraints; here regulation appears to *break* this circularity.

This breaking is apparent in the OA’s shift from viewing closure as a stable organisation described *in abstracto*, in favour of the notion of *organisational continuity*, reintroducing *diachronic* relations between different organisational configurations. As conceptualised by DiFrisco and Mossio (2020) to explain diachronic identity in complex life cycles, organisational continuity is defined by “the presence of a continuous causal process linking successive organizational regimes, irrespective of material and functional changes” (p. 1; see Mossio & Pontarotti, 2019). Two organisational configurations O_1_-at-t_1_ and O_2_-at-t_2_ are continuous when the latter causally depends on the former (cf. Bich & Skillings, 2024, p. 254, n. 4). This still entails “the conservation of a principle of *closure*” (DiFrisco & Mossio, 2020, p. 8), but in the much more generic sense that all organisationally continuous organisations instantiate closure despite differences. When DiFrisco and Mossio understand development simply as an organisationally continuous process where successive organisations differ, without producing numerically distinct individuals (as in fission or fusion; 10 ff.) What remains unspecified is the particular connection between stages proper to development as such.

Still, DiFrisco and Mossio’s work supports the idea that developmental purposiveness involves no intermediate stages: “an ontogenetic trajectory does not have to conserve anything in particular, neither structures nor functions” (p. 6), nor any *telos*. Bich and Skillings specify that development is not just a succession of structurally dependent organisations, but a *regulated* one, whereby regulation mediates transitions endowing them with a specific direction – so that, for instance, a caterpillar turns into a butterfly, not a frog. Moreover, development is not something that passively happens to a self-maintaining organism: “[t]o undergo development, this organization should also exhibit regulatory capabilities, that is, be able to determine *its own* processes of change” (Bich & Skillings, 2024, p. 256). Coherently, development should also be considered a reflexive activity realising self-determination through self-constraint. In what sense then is development purposive? If we “identify… the *telos* of the developing system in its current organization, rather than in a future state” (p. 251), unless we admit that development amounts to continuously “successfully failing” to achieve one’s goals^24^, we should assume that achieving a *telos* somehow equals to its own change or overcoming.

This raises major difficulties, especially given the reflexive nature of natural purposiveness. If purposiveness entails *self*-production, *self*-organisation, or *self*-constraint, development not only complicates the identification of a *self* (something that could be reflexive), but it seems to deny that life takes the form of a stable self at all (except perhaps momentarily, as a transient stabilising pattern of self-maintenance). The difficulties evaded by the minimal model, via positing an invariant self-maintaining organisation, now re-emerge. If developmental change prevents the identification of an organisation that can be both cause and effect of this activity, if not nominally, can teleology still be naturalised? Are organisational continuity and regulation enough to provide a principle of identity, and thus reflexivity? With these questions in mind, the next section turns to a set of critiques directed at the OA of development.

### Physiology vs. development: two different *telē*?

Important reservations towards the possibility of an OA of development have been voiced by Nuño de la Rosa (2010) and more recently by Rama (2026). Two lines of critique can be distinguished: (i) self-maintenance cannot account for development; (ii) self-maintenance and development have fundamentally different goals, respectively the *maintenance* of an organisation, and its *change* in function of a future organisation to be.

The first line of critique hinges on the observation that organisms are not capable of self-maintenance at all stages of development. As shown by Nuño de la Rosa (2010) in a detailed analysis of the causal relations involved in vertebrate ontogeny, the kind of functional integration enabling self-maintenance of the organism as a whole is only a *later* outcome of development. Organisms display a “double way of being” (p. 295): an early developmental mode, and a later self-maintaining mode. During the first phase, they alternately lose and gain essential features of full-fledged self-maintaining closure. A zygote is functionally integrated and with clear-cut boundaries, but cells and morphogenetic fields during cleavage and gastrulation are not, while individuality (impossibility of twinning) appears at gastrulation even without functional integration. Integration and boundaries re-emerge only partially during organogenesis and fully at the multicellular level once differentiation is complete and growth begins. Because essential features of closure are absent in early development, this does not depend on parts/whole relations: “early cleavage is mainly determined by molecular and physical mechanisms at the level of the individual cell, where cell-cell interactions play no role” (p. 302).

Nuño de la Rosa’s analysis challenges – well before Bich and Skillings’ paper! – the idea that the *telos* of biological organisation is present at each stage of development. Bich and Skillings’ response, though coherent with the OA, might appear at odds with common views on the matter: they simply “question the idea that the egg is the starting point of development” (p. 258). Cleavage, for instance, would not count as a stage of a developmental system, since such a system does not yet exist: “development starts when cells come together to form one integrated multicellular system – one organism – that then undergoes changes regulated at the level of the whole” (*ibidem*). Thus, the fertilised egg reproduces into many cells that only later transition to proper multicellular closure. Whereas for Nuño de la Rosa self-maintenance presupposes development, for Bich and Skillings development presupposes continuous self-maintenance.

While I do not mean to take a side here, I suggest that a possible conciliation between the two perspectives can be found in viewing the *whole life cycle* as a functionally integrated organised system. Drawing on Griesemer (2014), life cycles are (i) complex, involving the alternation of unicellular, multicellular, and intermediate organisations, and (ii) scaffolded, realised through regulated interactions both *between* organisations (e.g. parent-offspring, or pre-integrated cells) and *within* them (among cellular constituents, integrated cells etc.). An analogous approach has already been pursued by Nuño de la Rosa and colleagues in the study of reproductive agency and within- and between-organisms purposive relations (Cortés-García et al., 2024; Nuño de la Rosa, 2023; Nuño de la Rosa et al., 2021). Recent OA work on cross-generational functions and maintenance of stability through inheritance (Mossio & Pontarotti, 2019; Mossio & Saborido, 2016; Pontarotti, 2015, 2024) similarly supports such an organisational view of the life cycle, which subsumes non-self-maintaining stages of development under a larger self-maintaining system.

Shifting the focus to a larger functionally integrated system, however, does not eliminate the problem of change highlighted above. The second line of critique thus distinguishes between two forms of teleology. Nuño de la Rosa (2010) explicitly characterises developmental teleology as more “Aristotelian”, involving the actualisation of an organism in potency, and therefore, the identification of a goal with a future state-to-be (whereas self-maintenance proper would be more “Kantian,” defined by full-fledged functional integration). Rama (2025) further develops this distinction, particularly situating the OA within the recent comeback of organismal development within evolutionary biology (Jaeger, 2024; Moczek et al., 2011; Moss & Newman, 2016; Nadolski & Moczek, 2023; Nicholson, 2014; Nuño de la Rosa & Müller, 2021; Walsh, 2015, 2021). He argues that the OA, originally devised to account for *physiology* (what I have called the minimal model, which sees regulation as a principle of stability), is fundamentally ill-suited for the kind of teleology proper to development.

According to Rama (2025), what is teleological about development – and what a teleological view of development should account for – are *transitions* (relations between an organisation O_1_-at-t_1_ and an immediately successive O_2_-at-t_2_) and *trajectories* (relations between O_1_-at-t_1_ and a distant organisation O_3_-at-t_n_). As he argues, neither can be accounted for by self-maintenance alone, insofar as (i) the physiological requirements at one stage do not explain why an organisation transitions into another, and (ii) the present conditions of existence on which the organism exerts its causal influence are not, clearly, the end-goal of developmental trajectories. The contrast with the minimal model’s teleology is clear: the organism *as it is* cannot explain how the organism *will become*.

In Rama’s view, trajectories need not be fixed or predetermined; they can be *open-ended*, directed toward a range of possible states, though still constrained within a viable morphospace. If physiological changes happen among a repertoire of available configurations (e.g. switching between metabolic strategies depending on concentration of nutrients in the environment), development involves the generation of a possible repertoire among many. Thus, while physiology achieves of an invariant goal through varying means, development determines possible goals themselves. Rama concludes that a different organisational teleological theory is needed, particularly one integrating insights from evo-devo e.g. distinguishing between *efficient* OA constraints, and *specifying* developmental constraints (Stotz & Griffiths, 2017); or distinguishing between physiological and developmental regulation.

Here too, I do not mean to take sides – though I suspect Rama points in the right direction, especially regarding the integration of the OA with broader agential and evo-devo views. Rather, I take these critiques as an opportunity to re-examine the OA as a naturalising program. Nuño de la Rosa and Rama seem to call for a *splitting account* i.e. distinct theories for physiological and developmental purposiveness. As Rama writes, “[d]evelopment, maintenance, and reproduction can be presented as central goals that organisms pursue… To reduce one of these to another would lead to phenomena without adequate explanation” (2025, p. 31). Similarly, Delancey (2006) had called for a splitting account for intra-generational and cross-generational functions, to which Saborido et al. (2011) responded with a unified OA based on cross-generational self-maintenance. In general, the OA seems to pursue a *unifying* project, interpreting diverse biological phenomena through a naturalising Kantian view of purposiveness. By contrast, Nuño de la Rosa’s and Rama’s view are (explicitly or implicitly) more Aristotelian: “[t]eleological explanations postulate *end*-states, *future*-oriented goals to be achieved, or *final* causes that guide the global action of the system” (Rama, 2025, p. 3), in stark contrast with the OA’s commitment to present goals and temporal abstraction.

As Nahas and Sachs (2023) recently warned, the problem is not only *whether* teleology can be naturalised, but *which* teleology we seek to naturalise. When it comes to development (or any other biological phenomenon really), “it would need to be *argued*, rather than assumed, that we ought to prefer an Aristotelian over a Kantian conception of teleology” (p. 17). In this vein, Bich and Skillings explicitly reject the otherwise familiar idea that development is (or appears) Aristotelian-like because it is (or appears to be) a future-oriented process of actualisation. More fundamentally, the claim that developmental processes possess a distinct teleological profile, irreducible to other forms of functional and regulated change, might warrant further elaboration. For instance, on what basis do we distinguish switching between available functions in a repertoire, and developing new functions – particularly if certain functions and organisations show themselves to be “available” only *post facto*? Moreover, development (self-change) and physiology (self-maintenance) are not so easily distinguished in real life biology. The regeneration of a salamander’s limb involves both regulated (local) transitions and trajectories, but it generally counts as self-repair (Brockes & Gates, 2014). *Dyctostelium* amoebas can transition into functionally integrated multicellular “slugs” to self-preserve (Li & Purugganan, 2011). In humans, cognitive identity can be dramatically reorganised as an adaptive response to the environment. By extending the scope of self-maintenance to phenomena which *do not maintain any self*, the OA blurs the boundary between physiology and development.

Despite these important differences between splitting approaches and the unifying tendency of the OA, they both share similar difficulties regarding naturalisation, which the design / epigeneticity distinction highlights. Contemporary critics of agential views in philosophy of biology often retort that the seemingly purposive activities of organisms (epigeneticity) can be reductively explained by reference to their evolved properties (design) (DiFrisco & Gawne, 2025; Potter & Mitchell, 2024). As I am going to show, the OA (as well as other views of biological purposiveness) cannot so easily evade this criticism as long as it still assumes a strong interpretation of design to explain the goal-directedness of developmental changes.

## Discussion: whence goals?

As I discussed in § 3, a fundamental obstacle to teleological naturalism is the assumption that organisms *should* act in certain determinate ways, i.e. produce certain expected developmental outcomes (or one among many within a norm of reaction), which reduces epigeneticity to evolutionary design, and thus goal-directed activities to classical non-teleological adaptations. Both the OA and the other views of developmental purposiveness discussed above face this obstacle.

To be sure, neither Nuño de la Rosa nor Rama suggests that future-orientedness should be grounded on a teleonomic or “preformationist” notion of genetic program. Nonetheless, they remain reliant on other *dispositional* devices such as developmental constraints (Austin, 2017; Austin & Nuño de la Rosa, 2018). More generally, it seems that, to explain development – or any regulated change – as purposive, the outcome (or a range of possible outcomes) has to be somewhat specified in advance in a dispositional modality; dispositions explain purposive change. This, in turn, raises the question of how such dispositions are shaped in the first place, i.e. how goals are shaped.

The OA faces an analogous problem, displaced at the level of regulatory functions as *explanans* of change. If regulation establishes the organisms’ goals, but regulatory systems are decoupled from constitutive constraints i.e. their functioning is underdetermined by present circular relations, where do such goals come from? “[W]hat prescribes the normal range of activity of such a system? How can we determine how such a sub-system ought to work?” (Corti, 2022, p. 15).

OA literature sometimes points to the evolved nature of these regulatory systems (Bechtel & Bich, 2024; Bich & Bechtel, 2022b; Moreno & Mossio, 2015). Organisms appear formally self-determining, but they do so according to evolved norms embedded in the very structure of their constraints. In other words, a crucial feature of biological purposiveness – not its realisation, which is already secured by the circularity of biological organisation, but the specific organisational form it takes through regulated changes, enabling development, adaptive self-regulation and self-maintenance – still depends on evolution by natural selection. And insofar as it is only possible if regulated, the kind of circularity and integratedness proper to purposiveness would itself be a traditional adaptation (DiFrisco & Gawne, 2025; Huneman, 2019; Potter & Mitchell, 2024). Hence, the OA might not actually escape the reduction of epigeneticity to design envisioned in Sect. 3. Simply, it would shift the focus from genes or other dispositional devices to regulation as a stand-in for evolutionary design: regulation establishes goals *on behalf of* natural selection. If this is the case, if organisms realise goals which they do not ultimately contribute to shape, it is not clear at all what kind of *intrinsic* teleology the OA actually naturalises.

A way forward might be envisioned by considering the space the OA shares with recent evo-devo theories of organismal agency in today’s theoretical landscape (as Rama suggests we do). These approaches argue that organismal goals, while pursued and achieved by organisms at the ontogenetic scale, are nonetheless shaped by evolution – an evolution that it is itself enacted by organisms (Tahar, 2022; Walsh, 2006, 2015, 2021). Regulated self-maintenance at one scale would therefore rely on an inherited repertoire shaped at the larger, cross-generational scale by organisms themselves during organisms-enacted evolution (Mossio & Pontarotti, 2019; Sultan, 2019; Sultan et al., 2022; Walsh & Sultan, 2024). In other words, while we cannot fully account for organismal purposiveness by looking at individual organisms alone, this does not mean that organisms simply enact inherited adaptations, but rather that they are embedded into a temporally larger organised system (which might be tentatively identified with the lineage). Intrinsic natural purposiveness has a *historical* dimension (Montévil, 2020; Montévil & Mossio, 2020; Moreno & Mossio, 2015). That intrinsic purposiveness – being *cause and effect of itself* – extends beyond the ontogenetic scale was already suggested by Kant (CPJ, 371), but then largely forgotten by Kantian views of organisms (Gambarotto & Nahas, 2022, pp. 10–11). The OA offers important resources to reintegrate this idea in an evolutionary perspective.

On this view, an OA-informed teleological naturalism would not simply reduce design to epigeneticity, or vice versa. Rather, it would reconceptualise these two dimensions of purposiveness – what goals organisms have, and how they pursue them – as two *temporal* dimensions, the evolutionary and the (developmental/physiological) ontogenetic one. As many researchers have recently emphasised, the two scales do not correspond to two distinct kinds of causes (ultimate vs. proximate; Laland et al., 2011; Mayr, 1961) but are rather interpenetrated are mutually determining – phylogenesis and ontogenesis share a common logic. Organisms really are what they do – they embody the goals they pursue, which are nothing but organisation itself – but this self-directed reflexivity unfolds historically. This is a tentative suggestion, but not unfounded. The idea of applying self-causation or reciprocal-causation to evolution is not new, but it has been variously proposed in different programs that seek to overcome the gene-centrism and selectionism of the Modern Synthesis through an organism-centred view of evolutionary causation, such as Levins and Lewontin’s dialectical biology (Levins & Lewontin, 1985; Lewontin, 1983), Maturana and Mpodozis’ idea of natural drift (Maturana & Mpodozis, 2000; Mpodozis, 2022), or the recent Extended Evolutionary Synthesis project (Lala et al., 2014, 2015, 2024; Uller & Helanterä, 2017; Uller & Lala, 2019). An evolutionary perspective on organisational closure not only as a product, but also as a cause of evolution, might not only benefit evolutionary theory, but also offer the OA, and biological autonomy theory more generally, a way forward in its project of naturalising teleology.

To conclude, I have shown that the OA offers a solution to the long-standing problem of causal realisability of teleology, but at the price of a very restricted view of teleology itself. However, when *change* is taken seriously – change in organisation, goals, purposiveness itself – naturalising teleology based on parts/whole reflexivity is still problematic. The risk, in other words, is to assume that changes are functional to the maintenance of a whole which remains itself only heuristically. Moreover, the notions of design and epigeneticity, capturing respectively goals and goal-directed activities, highlight how insofar as we ultimately refer to Darwinian evolution to account for the seemingly purposive character of organisms, we are still only doing away with teleology – a risk still present for teleological naturalism. Whereas an evolutionary look at organisational teleology might shed light on the historical dimension of purposiveness, this would still require a more refined defence of teleological naturalism capable of accounting for change in purposiveness itself. To date, the OA constitutes an important and fruitful advancement in defence of an idea of teleology in contemporary biology and opens new frontiers of research. However, it also shows that the question of teleology is far from settled.

## Footnotes

1 I will use “teleology” to denote (causal) explanations that appeal to means, goals, as well as functions and norms, and “purposiveness” to denote alleged organismal properties accounted for by teleology. This terminology follows my use of Pluhar’s 1790 translation of Kant’s *Critique of the Power of Judgment*, where Kant’s *Naturzweck* is rendered as “natural purpose”, by which I hope to preserve conceptual consistency throughout the paper.

2 Even though recent works in the theory of biological autonomy have focused on regulation and changeability, the distinction I draw between the minimal and the regulative model is not historical, but theoretical. Specifically, it is a matter not of how biological organisation is modelled (on this, see Rider, 2025), but of how teleology is conceptualised. Mossio and Bich’s (2017) paper, initially published online in 2014, is the main work on teleology within the minimal model, whereas Bich’s (2024b) “organisational teleology 2.0” offers a conceptual basis for the regulative model.

3 Although Kant never speaks of “organisms” in the CPJ (Wolfe, 2010), but rather of “organised beings”, most scholars have taken Kant’s *Analytic of Teleological Judgment* to describe organisms (Huneman, 2024). A notable exception is Wilks (2023), who extends natural purposiveness to sub- and supra-organismal entities as well.

4 If my goal is to pull off a heist, and my accomplice asks why I am bringing a mask, I might answer: “it’s *part* of the plan!” Similarly, organismal parts are all traits at different levels of organisation – genes, proteins, cells, tissues, morphological patterns, behaviours etc.

5 Kant’s last point is particularly fascinating, as it includes not only what we call the “functional integration” between parts, but also what we might call the organism’s “adaptive plasticity,” by which “certain parts, on account of their deficiencies or impediments, form in an entirely new way so as to preserve… what is there,” (CPJ 372).

6 To be precise, Potter and Mitchell concede agency in behaviour alone, while they are critical of its application to development as well as to the alleged causal role of organisms in evolution. DiFrisco and Gawne instead believe that agency *tout court* is a failed concept without any possible research program.

7 To my knowledge, I am the first to contrast the OA with Teufel’s (2011) critique to teleological naturalism.

8 Teufel’s example of downstream whole-to-parts dependence is that of a “contraction in the global economy [that] *caused* tent cities to go up in California” (Teufel, 2011, p. 257). Even in this case, arguably, it is not the whole that produced a downstream effect on its parts (from global economy to tent cities), but only a part of the global economy itself (e.g. landlords making rent higher etc.). In fact, many would argue that blaming the “global economy” for the impoverishment of the lower class is an excuse to hide the responsibility of certain *parts* of this global economy! Thus, even downstream whole-to-parts causation might be reduced to parts-to-parts causation, as argued by DiFrisco and Gawne (2025).

9 For a recent discussion of Kant’s view of purposiveness as systematic unity, see Teufel 2025).

10 It is contentious whether etiological or selected-effects approaches genuinely naturalise teleology, or rather naturalise it away, reducing it to an epiphenomenon of evolutionary mechanics (Christensen & Bickhard, 2002; Mossio et al., 2009; Mossio & Saborido, 2016).

11 Note, my use of “epigeneticity” in this paper is strictly related to teleology and it expresses how activities are goal-directed via parts/whole relations. It bears no necessary connections with other “epigenetic” concepts such as “epigenetic landscape” (Waddington, 1957), “epigenetic control systems”(Nanney, 1958), or “epigenetic inheritance” (Jablonka & Lamb, 2010). Moreover, while it is historically opposed to preformationism (Casetta, 2020), epigeneticity *qua teleology* is also susceptible to a “preformationist” interpretation, i.e. teleonomy (see below). I thank the anonymous reviewer for bringing attention to this point.

12 An important disclaimer is in order. The relationship between teleology and evo-devo is a complex and all but obvious one. First, as Huneman himself notes (2017, pp. 385-386), research programs in evo-devo that focus instead on *physico-chemical* self-organisation do not necessarily refer to a purposive meaning of the “whole”. Second, the integration of teleological theories of biological agency and insights from evo-devo is still under debate (Newman, 2023; Walsh, 2021). Finally, as aptly pointed out by a reviewer, some of evo-devo’s *structuralist* foundations (e.g. focus on the *Bauplan* as developmental bias on variation; cf. Willmore, 2012) emerged in opposition to the teleological and functionalist language of Modern Synthetic adaptationism. Perhaps, a more paradigmatic case of teleological language in developmental biology might be found in eco-devo (Rama, 2021).

13 I thank the anonymous reviewer for raising this point.

14 For a discussion of normativity and viability, see Barandiaran (2025); Barandiaran & Egbert (2014).

15 Bich (2024b) has similarly called initial versions of the OA, which do not centre regulation, too “minimal” or “minimalist”. In my reading, the minimal model can and does include regulatory control, at least insofar as it serves the maintenance of certain organisational invariants. To the extent that it does not problematise organisational changes, even a regulatory “organisational teleology 2.0” might also be too “minimalist”. Elaborating an organisational teleology 3.0 will be the endeavour for a future work.

16 An alternative but equivalent definition takes processes as material entities undergoing transformation, avoiding splitting transformations (processes) from their outcomes (constraints).

17 For a discussion on organismal persistence, see Smith (2017).

18 This is only a conceptual point; identifying the temporal origin of a whole organism might not be so easy (Maienschein, 2011). Plus, the minimal model tells us nothing about the first coming-into-existence of a self-maintaining whole (via reproduction and development; see  5).

19 Unless of course C_1_ and C_2_ act at the same timescale *one after the other* on the same process.

20 Indeed, it is questionable that any entity can remain the same in this strict sense (except maybe for fundamental particles).

21 Note, not all conditions of existence count as goals. External factors like prey or shelter are crucial for survival but they are not produced by the organism; however, artefacts such as nest and hives might still be seen as subject to closure (Mossio & Pontarotti, 2019). Moreover, organisms do shape their own environmental conditions, making them *interactive* agents (Moreno & Mossio, 2015).

22 I thank the anonymous reviewer for highlighting this point.

23 To be fair, it might be useful to assume something like an organisational invariant even for complex systems – for instance, Barandiaran and Moreno take an organisational approach to neurocognitive functions based on the idea of regulation acting on behalf of a normative cognitive identity, a “self” (Barandiaran & Moreno, 2006; Moreno & Mossio, 2015, p. 187ff).

24 In this vein, Levin, (2023) states that “from the perspective of each embryonic stage, the prior stage has the incorrect anatomy” (p. 180; quoted in Rama, 2025, p. 13).
